# A Weighted Measurement Fusion Particle Filter for Nonlinear Multisensory Systems Based on Gauss–Hermite Approximation

**DOI:** 10.3390/s17102222

**Published:** 2017-09-28

**Authors:** Yun Li, Shu Li Sun, Gang Hao

**Affiliations:** 1 School of Electronic Engineering, Heilongjiang University, Harbin 150080, China; liyunhd@sina.com (Y.L.); haogang@hlju.edu.cn (G.H.); 2 School of Computer and Information Engineering, Harbin University of Commerce, Harbin 150001, China

**Keywords:** nonlinear system, weighted measurement fusion, Gauss–Hermite approximation, particle filter

## Abstract

We addressed the fusion estimation problem for nonlinear multisensory systems. Based on the Gauss–Hermite approximation and weighted least square criterion, an augmented high-dimension measurement from all sensors was compressed into a lower dimension. By combining the low-dimension measurement function with the particle filter (PF), a weighted measurement fusion PF (WMF-PF) is presented. The accuracy of WMF-PF appears good and has a lower computational cost when compared to centralized fusion PF (CF-PF). An example is given to show the effectiveness of the proposed algorithms.

## 1. Introduction

State estimation algorithms play an important part in automatic control, target tracking, navigation, fault diagnosis, and so on. However, it is difficult for single sensor to obtain accurate estimation and good fault tolerance, so multisensory fusion estimation technology was created to overcome this issue. There are two main kinds of fundamental estimation fusion structures: centralized fusion and distributed fusion. Centralized fusion combines measurements of all sensors into an augmented measurement, and then the data processing center outputs the fused state estimate. The advantage of centralized fusion is that there is no information loss and is ideal when all sensors are fully functional, so it is used as a comparative standard for other fusion algorithms. However, with a large number of sensors, the centralized fusion algorithm performs poorly in real time and is not reliable because of expensive computation due to augmented high-dimension measurements. In the sensor network, a large number of different types of sensors exist. If the data from the sensors are processed in a centralized way, the computational cost will be high. Especially in wireless sensor networks, large amounts of data from nodes make it difficult for decision centers to make timely decisions. In such cases, data compression must be used, and weighted measurement fusion (WMF) is one of these data compression methods [1,2,3].

Distributed fusion algorithms produce a fused estimate by combining local state estimates under a certain criterion in the fusion center [4,5,6,7]. Distributed fusion algorithms, such as the distributed fusion federated Kalman filter [8], the distributed weighted fusion estimation [9], and the distributed covariance intersection (CI) fusion estimation [10], are robust and flexible because of their parallel computing structures [3]. They are optimal for local application but are suboptimal for global use, as their accuracy is lower than the centralized fusion algorithm. WMF algorithms compress a high-dimension measurement to a low-dimension measurement under certain criterion [11,12,13], so the computational cost can be reduced when implementing particle filter (PF) based on the compressed measurement. For linear systems, the WMF algorithms are equivalent to centralized fusion [11,13], so they are also optimal in terms of least mean squares. In this paper, the WMF method will be studied given the abovementioned advantages.

Estimation fusion has formed a complete theory for linear systems over the past years. However, most systems have nonlinear parts. For example, the measurement functions, in sensor models, of most tracking systems are established under spherical coordinates, and are strongly nonlinear when the states are estimated under Cartesian coordinates [14,15]. For nonlinear systems, fusion algorithms, which are achieved by the Taylor series, based on the Extended Kalman filter (EKF) are commonly used [16,17,18,19]. These algorithms are simple since they can be converted into linear systems. However, they result in large estimation bias and even lead to filtering divergence due to the amount of information being omitted. Many nonlinear fusion approaches have been presented, including random set, artificial neural networks, fuzzy logic, rough set, dempster-shafer, and other non-probabilistic approaches [20,21]. These methods fuse information and compress data. Given the amount of information lost, they are usually suboptimal. 

Nonlinear filtering algorithms, based on the Bayesian estimation framework and sample approximation, have been widely studied over the past decades, including the Extended Kalman filter (EKF), Unscented Kalman Filter (UKF) [22,23], Cubature Kalman Filter (CKF), and the particle filter (PF) [24]. These algorithms are effective for nonlinear filtering problems with a single sensor [25,26,27,28]. In Hao et al. [29], we presented a weighted measurement fusion UKF (WMF-UKF) via Taylor series and UKF, which can universally handle nonlinear fusion problems. However, this algorithm needs to calculate the coefficients of a Taylor series expansion at every moment, so it is expensive and leads to slow convergence if the wrong expansion location is used.

PF can solve the estimation problem for nonlinear non-Gaussian systems and has high accuracy with an adequate number of sampling points. However, a large number of sampling points results in a huge computational burden, especially for a multisensor centralized fusion estimator. Gauss–Hermite approximation [30,31,32,33] can approximate most nonlinear functions with some sampling points and has an excellent fitting effect. We first proposed a weighted measurement fusion based on Gauss–Hermite approximation and weighted least squares. Next, a nonlinear weighted measurement fusion particle filter (WMF-PF) is presented by combining the fusion algorithm with PF for nonlinear multisensory systems. The proposed algorithm handles the nonlinear fusion problem with any noise and reduces the computational cost compared to centralized fusion PF. Moreover, it overcomes the shortcomings found in Hao et al. [29]. WMF-PF provides an effective compression method for nonlinear multisensory systems, and has potential applications in target tracking [34], communication, and massive data processing. 

## 2. Problem Formulation

In order to facilitate the description of the algorithm, we used the scalar systems as an example. Consider the scalar nonlinear systems with *L* sensors:

(1) x(k+1)=f(x(k),k)+w(k)

(2) $$z_{}^{\left(j\right)}(k)=h_{}^{\left(j\right)}(x(k),k)+v_{}^{\left(j\right)}(k),\;j=1,2,\cdots ,L$$

where f(⋅,⋅)∈ℜ is the process function, x(k)∈ℜ is the scalar state at time k, $h^{\left(j\right)}(\cdot ,\cdot )\in ℜ$ is the measurement function of the *j*th sensor, $z^{\left(j\right)}(k)\in ℜ$ is the measurement of the *j*th sensor, $w(k)∼p_{w_{k}^{}}(\cdot )$ ($p_{*}(\cdot )$ is the probability density function) is the process noise, and $v_{}^{\left(j\right)}(k)∼p_{v_{k}^{\left(j\right)}}^{}(\cdot )$ is the measurement noise of the *j*th sensor. w(k) and $v_{}^{\left(j\right)}(k)$ are uncorrelated white noises with zero mean and variances, $\sigma_{w}^{2}$ and $\sigma_{vj}^{2}$, i.e.,

(3) $$Ε\left\{\left[\begin{array}{c} w(t)\\ v^{\left(j\right)}(t) \end{array}\right]\left[\begin{array}{cc} w^{T}(k) & v^{\left(l\right)}{}_{}^{T}(k) \end{array}\right]\right\}=\left[\begin{array}{cc} \sigma_{w}^{2} & 0\\ 0 & \sigma_{vj}^{2}\delta_{jl} \end{array}\right]\delta_{tk}$$

where E denotes the mathematical expectation, the superscript T denotes the transpose, and $\delta_{tk}$ and $\delta_{jl}$ are the Kronecker delta functions, i.e., $\delta_{tt}=1$ and $\delta_{tk}=0(t\neq k)$.

Assumption 1:  The f(⋅,⋅), $h^{\left(j\right)}(\cdot ,\cdot )$, $p_{w_{k}^{}}(\cdot )$, and $p_{v_{k}^{\left(j\right)}}^{}(\cdot )$ are known.

Assumption 2:  The state x(k) is bounded.

For the systems in Equations (1) and (2), the augmented measurement equation of the centralized fusion system (CFS) is given a

(4) $$z^{\left(0\right)}(k)=h_{}^{\left(0\right)}(x(k),k)+v^{\left(0\right)}(k)$$

where

(5) $$z^{\left(0\right)}(k)={\left[z_{}^{\left(1\right)}(k),z_{}^{\left(2\right)}(k),\cdots ,z_{}^{\left(L\right)}(k)\right]}^{T}$$

(6) $$h^{\left(0\right)}(x(k),k)={\left[h_{}^{\left(1\right)}(x(k),k),h_{}^{\left(2\right)}(x(k),k),\cdots ,h_{}^{\left(L\right)}(x(k),k)\right]}^{T}$$

(7) $$v^{\left(0\right)}(k)={\left[v_{}^{\left(1\right)}(k),v_{}^{\left(2\right)}(k),\cdots ,v_{}^{\left(L\right)}(k)\right]}^{T}$$

and the covariance matrix of $v^{\left(0\right)}(k)$ is given as

(8) $$R^{\left(0\right)}=\mathrm{diag}(\sigma_{v1}^{2},\sigma_{v2}^{2},\cdots ,\sigma_{vL}^{2})$$

where diag(·) denotes a diagonal matrix.

For the systems in Equations (1) and (4), we obtained the centralized fusion PF combined with the particle filter. However, Equation (4), with a high dimension, will result in high computational costs, particularly in massive sensor networks. Therefore, it is important to find the equivalent or approximate fusion methods to reduce the computational cost.

**Lemma 1.**  *For the systems in Equations (1) and (2), if there are linear relationships between measurement functions* $h_{}^{\left(j\right)}(x(k),k)$, j=1,2,⋯,L, *that is to say*, *there is a intermediary function* $h(x(k),k)\in {ℜ}^{p\times 1}$ *that satisfies* $h_{}^{\left(j\right)}(x(k),k)=H_{}^{\left(j\right)}(k)h(x(k),k)$ *with matrix* $H_{}^{\left(j\right)}(k)\in {ℜ}^{1\times p}$, *the compressed measurement function of the weighted measurement fusion system (WMFS) is given as [29]*

(9) $$z^{\left(I\right)}(k)=H^{\left(I\right)}(k)h(x(k),k)+v^{\left(I\right)}(k)$$

(10) $$z^{\left(I\right)}(k)={\left[M^{T}(k)R^{(0)-1}M(k)\right]}^{-1}M^{T}(k){\left(R^{\left(0\right)}\right)}_{}^{-1}z^{\left(0\right)}(k)$$

(11) $$v^{\left(I\right)}(k)={\left[M^{T}(k)R^{(0)-1}M(k)\right]}^{-1}M^{T}(k){\left(R^{\left(0\right)}\right)}_{}^{-1}v^{\left(0\right)}(k)$$

(12) $$R^{\left(0\right)}{}_{}^{-1}={\left(R^{\left(0\right)}\right)}^{-1}$$

The covariance matrix of $v^{\left(I\right)}(k)$ is computed by

(13) $$R^{\left(I\right)}(k)={\left[M^{T}(k)R^{(0)-1}M(k)\right]}^{-1}$$

where M(k), with full-column rank, and $H_{}^{\left(Ι\right)}(k)$, with full-row rank, are the full rank decomposition matrices of matrix $H^{\left(0\right)}(k)={\left[H_{}^{(1)T}(k),\cdots ,H_{}^{(L)T}(k)\right]}^{T}$, that is,

(14) $$H^{\left(0\right)}(k)=M(k)H_{}^{\left(Ι\right)}(k)$$

which can be computed by Hermite canonical form [29].

We assumed that the statistics of w(k) and $v_{}^{\left(j\right)}(k)$, j=1,2,⋯,L are known. In fact, we obtained the noise statistics through identification [35,36,37]. For time-invariant systems, we identified them offline and obtained the optimal weighted measurement fusion algorithms. For time-varying systems, we identified them online and obtained the asymptotic optimal adaptive weighted measurement fusion algorithm. 

In order to determine the weighted measurement fusion based on the probabilistic method, we needed to know two things: the relationships among the noise statistics of all the sensors and the relationships among the measurement functions of all sensors. For systems with additive noises, if we knew the relationships between the measurement functions, the relationships among the measurement noise statistics could be determined. If a linear relationship exists among the measurement functions, whether the measurement functions themselves are linear or not, we could use the least square method to find the optimal compressed measurement. If a nonlinear relationship exists among the measurement functions, we could use intermediary functions to achieve the optimal compressed measurement. Unfortunately, finding the intermediary function is difficult because of the complexity and diversity of nonlinear functions. 

For example, there are four sensors and their measurement functions are as follows:

(15) $$z_{}^{\left(1\right)}(k)=1+x(k)+x_{}^{2}(k)+v_{}^{\left(1\right)}(k),\;z_{}^{\left(2\right)}(k)=2+x(k)+2x_{}^{2}(k)+x_{}^{3}(k)+v_{}^{\left(2\right)}(k),\;z_{}^{\left(3\right)}(k)=3+2x(k)+3x_{}^{2}(k)+x_{}^{3}(k)+v_{}^{\left(3\right)}(k),\;z_{}^{\left(4\right)}(k)=4+x(k)+4x_{}^{2}(k)+3x_{}^{3}(k)+v_{}^{\left(4\right)}(k)$$

where the variances of $v_{}^{\left(j\right)}(k),\;j=1\cdots 4$ are $R_{}^{\left(1\right)}=1$, $R_{}^{\left(2\right)}=2$, $R_{}^{\left(3\right)}=3$, and $R_{}^{\left(4\right)}=4$. 

Set $h(x(k),k)={\left[\begin{array}{cccc} 1 & x(k) & x_{}^{2}(k) & x_{}^{3}(k) \end{array}\right]}_{}^{T}$, then Equation (15) can be written as

(16) $$z_{}^{\left(1\right)}(k)=[\begin{array}{cccc} 1 & 1 & 1 & 0 \end{array}]h(x(k),k)+v_{}^{\left(1\right)}(k),\;z_{}^{\left(2\right)}(k)=[\begin{array}{cccc} 2 & 1 & 2 & 1 \end{array}]h(x(k),k)+v_{}^{\left(2\right)}(k),\;z_{}^{\left(3\right)}(k)=[\begin{array}{cccc} 3 & 2 & 3 & 1 \end{array}]h(x(k),k)+v_{}^{\left(3\right)}(k),\;z_{}^{\left(4\right)}(k)=[\begin{array}{cccc} 4 & 1 & 4 & 3 \end{array}]h(x(k),k)+v_{}^{\left(4\right)}(k)$$

Let

(17) $$H^{\left(0\right)}=\left[\begin{array}{cccc} 1 & 1 & 1 & 0\\ 2 & 1 & 2 & 1\\ 3 & 2 & 3 & 1\\ 4 & 1 & 4 & 3 \end{array}\right]=MH_{}^{\left(Ι\right)}=\left[\begin{array}{cc} 1 & 1\\ 2 & 1\\ 3 & 2\\ 4 & 1 \end{array}\right]\left[\begin{array}{cccc} 1 & 0 & 1 & 1\\ 0 & 1 & 0 & -1 \end{array}\right]$$

where M is full-column rank, and $H_{}^{\left(Ι\right)}$ is full-row rank. According to the weighted least squares criterion, the fused measurement function is

(18) $$z^{\left(I\right)}(k)=H^{\left(I\right)}(k)h(x(k),k)+v^{\left(I\right)}(k)$$

where $z^{\left(I\right)}(k)={\left[M^{T}(k){\left(R^{\left(0\right)}\right)}_{}^{-1}M(k)\right]}^{-1}M^{T}(k){\left(R^{\left(0\right)}\right)}_{}^{-1}z^{\left(0\right)}(k)=$$\left[\begin{array}{cccc} -0.3286 & 0.1000 & -0.0429 & 0.3143\\ 0.8571 & 0 & 0.2857\; & -0.4286 \end{array}\right]z^{\left(0\right)}(k)$, and the covariance of $v^{\left(I\right)}(k)$ is $R^{\left(I\right)}(k)={\left[M^{T}(k)R^{(0)-1}M(k)\right]}^{-1}=$$\left[\begin{array}{cc} 0.5286 & -0.8571\\ -0.8571 & 1.7143 \end{array}\right]\;$. The measurement function of WMF is two-dimensional, and that of the centralized fusion system is four-dimensional, so the WMF can compress measurement dimensions effectively and reduce the computational cost.

In the next section, we provide an approximation method to solve this problem.

## 3. Gauss–Hermite Approximation

**Lemma 2.**  *Let* y(x) *be a determined function* [30]. *Assuming that there is an ensemble of* S *points* $\left\{x_{i}^{},\;i=1,\cdots ,S\right\}$ *distributed uniformly in the interval* [a, b], *there exists a point* $y_{i}^{}$ *such that* $y_{i}^{}=y(x_{i}^{})$ *for each point* $x_{i}^{}$. *Then, the approximation function* $\bar{y}(x)$ *of* y(x), *by Gauss–Hermite folding reads*

(19) $$\bar{y}(x)=\frac{1}{\gamma \sqrt{\pi }}\sum_{i=1}^{S}y_{i}^{}\Delta x_{i}^{}\exp\left\{-{\left(\frac{x-x_{i}^{}}{\gamma }\right)}_{}^{2}\right\}f_{n}^{}\left(\frac{x-x_{i}^{}}{\gamma }\right)$$

where γ is a coefficient, $\Delta x_{i}^{}=\frac{1}{2}(x_{i+1}^{}-x_{i-1}^{})$, and $f_{n}^{}(u)$ is the correction polynomials which can be decomposed into a series of Hermite polynomials:

(20) $$f_{n}^{}(u)=\sum_{i=1}^{n}C_{i}^{}H_{i}^{}(u)$$

(21) $$C_{i}^{}=\frac{1}{2_{}^{i}i!}H_{i}^{}(0)$$

where $H_{i}^{}(u)={\left(-1\right)}_{}^{i}e_{}^{u_{}^{2}}{\left(e_{}^{-u_{}^{2}}\right)}_{}^{\left(i\right)}$ represents Hermite polynomials [31], and $H_{i}^{}(0)$ is defined as

(22) $$H_{i}^{}(0)=\{\begin{array}{cc} \begin{array}{l} 1\\ 2_{}^{n}{\left(-1\right)}_{}^{n}(2n-1)!!\\ 0 \end{array} & \begin{array}{l} i=0\\ i=2n\\ i=2n+1 \end{array} \end{array}$$

From Equations (21) and (22), we obtain

(23) $$C_{i}^{}=\{\begin{array}{ll} 1 & i=0\\ {\left(-1\right)}_{}^{n}\frac{(2n-1)!!}{2_{}^{n}(2n)!} & i=2n\\ 0 & i=2n+1 \end{array}$$

See the detailed proof in the literature [30,31,32,33].

**Remark 1.**  *Although* γ *is an arbitrary coefficient* *related to* $\Delta x_{i}^{}$, *it was found that* $\bar{y}(x)$ *approximates better when* γ=1 *and* n=2 *or 4, for most elementary functions* [30]. *Thus, we used* γ=1 *and* n=2 *in this paper, which yielded*

(24) $$\bar{y}(x)=\frac{1}{\sqrt{\pi }}\sum_{i=1}^{S}y_{i}^{}\Delta x_{i}^{}e_{}^{-{\left(x-x_{i}^{}\right)}_{}^{2}}(1.5-{\left(x-x_{i}^{}\right)}_{}^{2})$$

## 4. Universal WMF Based on Gauss–Hermite Approximation

If the function $\sum_{i=1}^{S}e_{}^{-{\left(x-x_{i}^{}\right)}_{}^{2}}(1.5-{\left(x-x_{i}^{}\right)}_{}^{2})$ in Equation (24) is seen as the intermediary function h(x(k),k), and $\frac{1}{\sqrt{\pi }}\sum_{i=1}^{S}y_{i}^{}\Delta x_{i}^{}$ is seen as $H_{}^{\left(j\right)}(k)$ in Lemma 2, then we can obtain the linear relationships among the measurement functions $h_{}^{\left(j\right)}(x(k),k)$ and obtain the following theorem using Lemma 2.

Based on Lemmas 1 and 2, we can obtain the following Theorem 1.

**Theorem 1.**  For the systems in Equations (1) and (2), and from Equation (24), the approximate measurement equation of the weighted measurement fusion system is

(25) $${\bar{z}}_{}^{\left(I\right)}(k)={\bar{H}}^{\left(I\right)}\left(\begin{array}{c} e_{}^{-{\left(x-x_{1}^{}\right)}_{}^{2}}(1.5-{\left(x-x_{1}^{}\right)}_{}^{2})\\ e_{}^{-{\left(x-x_{2}^{}\right)}_{}^{2}}(1.5-{\left(x-x_{2}^{}\right)}_{}^{2})\\ \vdots \\ e_{}^{-{\left(x-x_{S}^{}\right)}_{}^{2}}(1.5-{\left(x-x_{S}^{}\right)}_{}^{2}) \end{array}\right)+{\bar{v}}_{}^{\left(I\right)}(k)$$

We denoted

(26) $${\bar{H}}^{\left(0\right)}=\frac{1}{\sqrt{\pi }}\left(\begin{array}{cccc} y_{1}^{\left(1\right)}\Delta x_{1}^{\left(1\right)} & y_{2}^{\left(1\right)}\Delta x_{2}^{\left(1\right)} & \cdots & y_{S}^{\left(1\right)}\Delta x_{S}^{\left(1\right)}\\ y_{1}^{\left(2\right)}\Delta x_{1}^{\left(2\right)} & y_{2}^{\left(2\right)}\Delta x_{2}^{\left(2\right)} & \cdots & y_{S}^{\left(2\right)}\Delta x_{S}^{\left(2\right)}\\ \vdots & \vdots & \cdots & \vdots \\ y_{1}^{\left(L\right)}\Delta x_{1}^{\left(L\right)} & y_{2}^{\left(L\right)}\Delta x_{2}^{\left(L\right)} & \cdots & y_{S}^{\left(L\right)}\Delta x_{S}^{\left(L\right)} \end{array}\right)$$

(27) $${\bar{z}}^{\left(I\right)}(k)={\left[{\bar{M}}^{T}R^{(0)-1}\bar{M}\right]}^{-1}{\bar{M}}^{T}R^{(0)-1}z^{\left(0\right)}(k)$$

(28) $${\bar{v}}^{\left(I\right)}(k)={\left[{\bar{M}}^{T}R^{(0)-1}\bar{M}\right]}^{-1}{\bar{M}}^{T}R^{(0)-1}v^{\left(0\right)}(k)$$

The covariance matrix of $v^{\left(I\right)}(k)$ is given by

(29) $${\bar{R}}^{\left(I\right)}={\left[{\bar{M}}^{T}R^{(0)-1}\bar{M}\right]}^{-1}$$

where $y_{k}^{\left(m\right)}$(k=1,⋯,S) is the *k*th sample point of the *m*th sensor, S is the sample point number, $\bar{M}$ and ${\bar{H}}^{\left(I\right)}$ are the full rank decomposition matrices of ${\bar{H}}^{\left(0\right)}$, $\bar{M}\in R_{}^{L\times r}$ is full-column rank, ${\bar{H}}_{}^{\left(Ι\right)}\in R_{}^{r\times S}$ is full-row rank, $r=\mathrm{rank}({\bar{H}}^{\left(0\right)})$, and r≤min{L, S}).

An approximate intermediary function,

(30) $$h(x(k),k)=\left(\begin{array}{c} e_{}^{-{\left(x-x_{1}^{}\right)}_{}^{2}}(1.5-{\left(x-x_{1}^{}\right)}_{}^{2})\\ e_{}^{-{\left(x-x_{2}^{}\right)}_{}^{2}}(1.5-{\left(x-x_{2}^{}\right)}_{}^{2})\\ \vdots \\ e_{}^{-{\left(x-x_{S}^{}\right)}_{}^{2}}(1.5-{\left(x-x_{S}^{}\right)}_{}^{2}) \end{array}\right)$$

has been constructed using the Gauss–Hermite approximation method in Lemma 2. It establishes some linear relationships between local measurement functions. Therefore, it solves the restriction that the measurement functions must be linear relationships in Lemma 1.

From the above theorem, we can see that, if there is a nonlinear system with *L* sensors, the centralized fusion system in Equations (1) and (4) has a measurement function with *L* dimension and the WMF system in Equations (1) and (25) has a measurement function with *r* dimension. Because $r=\mathrm{rank}({\bar{H}}^{\left(0\right)})$ and r≤min{L, S}, the computational cost of the WMF filter is less than that of the centralized fusion filter. After the sample point number S is determined, the compression efficiency of the algorithm is significantly improved if there are a large number of sensors (L>>S and *S* is a constant).

## 5. WMF-PF Based on Gauss–Hermite Approximation

In this section, we propose a WMF-PF algorithm based on the WMF system in Equations (1) and (25) with a low-dimension measurement. 

### 5.1. WMF-PF Algorithm

The computational procedure of WMF-PF is given as follows:

1. Initialization: ${\hat{x}}_{}^{\left(Ι)(i\right)}(0|0)∼p_{x_{0}^{}}^{}(x_{0}^{}),\;i=1,\cdots ,N_{s}^{}$;

2. State prediction particles:

(31) $${\hat{x}}_{}^{\left(Ι)(i\right)}(k|k-1)=f({\hat{x}}_{}^{\left(Ι)(i\right)}(k-1|k-1),k-1)+\zeta_{}^{\left(Ι)(i\right)}(k-1)$$

where $\zeta_{}^{\left(Ι)(i\right)}(k-1)$ is random number with the same distribution of the process noise w(k);

3. Measurement prediction particles:

(32) $${\hat{z}}_{}^{\left(Ι)(i\right)}(k|k-1)={\bar{H}}^{\left(I\right)}h({\hat{x}}_{}^{\left(Ι)(i\right)}(k|k-1),k)$$

4. The importance weight:

(33) $$\omega_{k}^{\left(Ι)(i\right)}=\frac{1}{N_{s}^{}}p({\hat{z}}_{}^{\left(Ι)(i\right)}(k|k-1)|{\hat{x}}_{}^{\left(Ι)(i\right)}(k|k-1))$$

that is,

(34) $$\omega_{k}^{\left(Ι)(i\right)}=\frac{1}{N_{s}^{}}p_{v_{k}^{\left(Ι\right)}}^{}({\bar{z}}_{}^{\left(Ι\right)}(k)-{\hat{z}}_{}^{\left(Ι)(i\right)}(k|k-1))$$

where ${\bar{z}}_{}^{\left(Ι\right)}(k)$ is computed by Equation (27), and ${\bar{\omega }}_{k}^{\left(Ι)(i\right)}$ is given by

(35) $${\bar{\omega }}_{k}^{\left(Ι)(i\right)}=\frac{\omega_{k}^{\left(Ι)(i\right)}}{\sum_{i=1}^{N}\omega_{k}^{\left(Ι)(i\right)}}$$

5. Filtering:

(36) $${\hat{x}}_{}^{\left(Ι\right)}(k|k)=\sum_{i=1}^{N_{s}^{}}{\bar{\omega }}_{k}^{\left(Ι)(i\right)}{\hat{x}}_{}^{\left(Ι)(i\right)}(k|k-1)$$

and the filtering variance matrix is computed by

(37) $$P_{}^{\left(Ι\right)}(k|k)\approx \sum_{i=1}^{N_{s}^{}}{\bar{\omega }}_{k}^{\left(Ι)(i\right)}{\left({\hat{x}}_{}^{\left(Ι)(i\right)}(k|k-1)-{\hat{x}}_{}^{\left(Ι)(i\right)}(k|k)\right)}^{2}$$

6. Resampling: In this paper, systematic sampling is used as the resampling method, i.e.,

(38) $$u_{i}^{}=\frac{(i-1)+r}{N},\;r∼U[0,1],\;i=1,\cdots ,N_{s}^{}$$

If $\sum_{j=1}^{m-1}{\bar{\omega }}_{k}^{\left(Ι)(j\right)}<u_{i}^{}\leq \sum_{j=1}^{m}{\bar{\omega }}_{k}^{\left(Ι)(j\right)}$, we directly copied m particles as the resampling particles ${\hat{x}}_{}^{\left(Ι)(i\right)}(k|k)$.

We then returned to Step 2 and reiterated.

**Figure 1:**
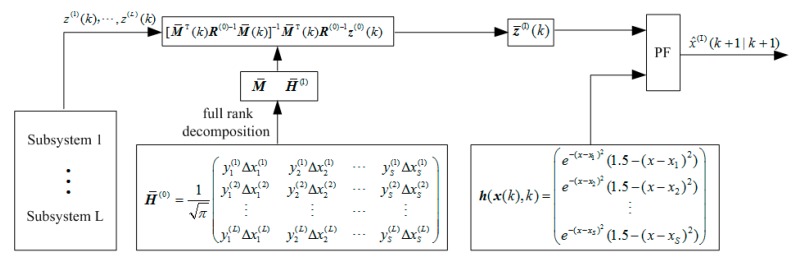
Flow chart of the weighted measurement fusion particle filter (WMF-PF) algorithm.

The flow chart of the WMF-PF algorithm is shown in Figure 1.

### 5.2. Time Complexity Analysis

From Equations (31)–(38), we found that the time complexity is determined by Equation (32). From Equation (32), the time complexity of centralized fusion PF (CF-PF) is $O(L_{}^{2})$ and that of WMF-PF is $O(r_{}^{2})$. Because r≤min{L, S}, the time complexity of WMF-PF is less than that of CMF-PF. Particularly, when there are a large number of sensors (L>>S), the computational cost can be substantially reduced.

## 6. Simulation Examples

### 6.1. Model Description

Let us consider a classical 10-sensor nonlinear system [38]:

(39) $$x(k)=\frac{x(k-1)}{4}+x(k-1)/\left(1+x{\left(k-1\right)}_{}^{2}\right)+2\cos(0.5(k-1))+w(k)$$

(40) $$z_{}^{\left(j\right)}(k)=h_{}^{\left(j\right)}\left(x(k),k\right)+v_{}^{\left(j\right)}(k),\;j=1,\cdots ,10$$

Taking observability into account, the sensors selected were single-valued functions within the range of x(k):

(41) $$h_{}^{\left(1\right)}\left(x(k),k\right)=0.8x(k)+v_{}^{\left(1\right)}(k)\;h_{}^{\left(2\right)}\left(x(k),k\right)=1.2x(k)+v_{}^{\left(2\right)}(k)\;h_{}^{\left(3\right)}\left(x(k),k\right)=\exp(x(k)/3)+v_{}^{\left(3\right)}(k)\;h_{}^{\left(4\right)}\left(x(k),k\right)=1.2\exp(x(k)/3)+v_{}^{\left(4\right)}(k)\;h_{}^{\left(5\right)}\left(x(k),k\right)=0.05x{\left(k\right)}_{}^{3}+v_{}^{\left(5\right)}(k)\;h_{}^{\left(6\right)}\left(x(k),k\right)=0.06x{\left(k\right)}_{}^{3}+v_{}^{\left(6\right)}(k)\;h_{}^{\left(7\right)}\left(x(k),k\right)=5\sin(0.1\pi x(k))+v_{}^{\left(7\right)}(k)\;h_{}^{\left(8\right)}\left(x(k),k\right)=6\sin(0.1\pi x(k))+v_{}^{\left(8\right)}(k)\;h_{}^{\left(9\right)}\left(x(k),k\right)=5\arctan(0.1\pi x(k))+v_{}^{\left(9\right)}(k)\;h_{}^{\left(10\right)}\left(x(k),k\right)=6\arctan(0.1\pi x(k))+v_{}^{\left(10\right)}(k)$$

The w(k)~U(0, 1) is uniformly distributed, and $v_{}^{\left(j\right)}(k)$, j=1,⋯,10 are uncorrelated Gaussian noises with variance $\sigma_{vj}^{2}={\left(0.5+0.01j\right)}_{}^{2}$. The initial state is x(0)=0.

### 6.2. Gauss–Hermite Approximation

**Figure 2:**
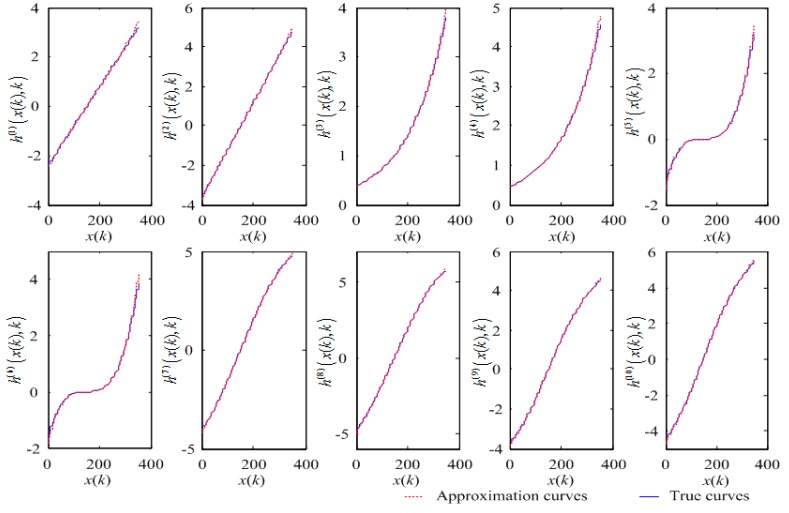
Approximation curves of nonlinear functions.

Because the state x(k) ranges from –3 to 4, we chose S=10 sampling points ($x_{i}^{}=-4,\;-3,\;\cdots ,\;5$) to approximate, and the corresponding coefficients were γ=1, $\Delta x_{i}^{}=1$, and n=2. Their mean square errors (MSEs) are shown in Table 1, and the approximation curves are shown in Figure 2. 

To compare with other approximate methods, we introduced the approximation method using a McLaughlin series. We used a third-order McLaughlin series to approximate the nonlinear functions $h_{}^{\left(j\right)}\left(x(k),k\right)$. The MSEs of the approximation algorithm, using a McLaughlin series, are also shown in Table 1. From Table 1, we can see that the MSEs of the approximation algorithm using a McLaughlin series are larger than that when using Gauss–Hermite approximation.

### 6.3. Estimation Using WMF-PF

From the above experiments, we see that the approximation effect is good when using the above coefficients. Next, we established the measurement equation and fusion matrix of WMF. As the intermediary function h(x(k),k) is ${\left(\begin{array}{ccc} e_{}^{-{\left(x-x_{1}^{}\right)}_{}^{2}}(1.5-{\left(x-x_{1}^{}\right)}_{}^{2}), & \cdots , & e_{}^{-{\left(x-x_{10}^{}\right)}_{}^{2}}(1.5-{\left(x-x_{10}^{}\right)}_{}^{2}) \end{array}\right)}_{}^{T}$, from Equations (24) and (25), the coefficient matrices ${\bar{H}}_{}^{\left(0\right)}$, and its full rank decomposition matrices $\bar{M}$ and ${\bar{H}}_{}^{\left(Ι\right)}$, can be computed as follows:

(42) $${\bar{H}}_{}^{\left(0\right)}=\left[\begin{array}{cccccccccc} -1.8054 & -1.3541 & -0.9027 & -0.4514 & 0 & 0.4514 & 0.9027 & 1.3541 & 1.8054 & 2.2568\\ -2.7081 & -2.0311 & -1.3541 & -0.6770 & 0 & 0.6770 & 1.3541 & 2.0311 & 2.7081 & 3.3851\\ 0.1487 & 0.2076 & 0.2897 & 0.4043 & 0.5642 & 0.7874 & 1.0989 & 1.5336 & 2.1403 & 2.9871\\ 0.1785 & 0.2491 & 0.3476 & 0.4851 & 0.6770 & 0.9449 & 1.3187 & 1.8404 & 2.5684 & 3.5845\\ -1.8054 & -0.7617 & -0.2257 & -0.0282 & 0 & 0.0282 & 0.2257 & 0.7617 & 1.8054 & 3.5262\\ -2.1665 & -0.9140 & -0.2708 & -0.0339 & 0 & 0.0339 & 0.2708 & 0.9140 & 2.1665 & 4.2314\\ -2.6829 & -2.2822 & -1.6581 & -0.8717 & 0 & 0.8717 & 1.6581 & 2.2822 & 2.6829 & 2.8209\\ -3.2195 & -2.7386 & -1.9897 & -1.0461 & 0 & 1.0461 & 1.9897 & 2.7386 & 3.2195 & 3.3851\\ -2.5350 & -2.1321 & -1.5825 & -0.8587 & 0 & 0.8587 & 1.5825 & 2.1321 & 2.5350 & 2.8319\\ -3.0420 & -2.5585 & -1.8990 & -1.0304 & 0 & 1.0304 & 1.8990 & 2.5585 & 3.0420 & 3.3983 \end{array}\right]\;\bar{M}=\left[\begin{array}{ccccc} -1.8054 & -1.3541 & -0.9027 & -0.4514 & 0\\ -2.7081 & -2.0311 & -1.3541 & -0.6770 & 0\\ 0.1487 & 0.2076 & 0.2897 & 0.4043 & 0.5642\\ 0.1785 & 0.2491 & 0.3476 & 0.4851 & 0.6770\\ -1.8054 & -0.7617 & -0.2257 & -0.0282 & 0\\ -2.1665 & -0.9140 & -0.2708 & -0.0339 & 0\\ -2.6829 & -2.2822 & -1.6581 & -0.8717 & 0\\ -3.2195 & -2.7386 & -1.9897 & -1.0461 & 0\\ -2.5350 & -2.1321 & -1.5825 & -0.8587 & 0\\ -3.0420 & -2.5585 & -1.8990 & -1.0304 & 0 \end{array}\right]\;{\bar{H}}_{}^{\left(Ι\right)}=\left(\begin{array}{cccccccccc} 1 & 0 & 0 & 0 & 0 & 0 & 0 & 0 & -1 & -6.0696\\ 0 & 1 & 0 & 0 & 0 & 0 & 0 & -1 & 0 & 15.5971\\ 0 & 0 & 1 & 0 & 0 & 0 & -1 & 0 & 0 & -21.6922\\ 0 & 0 & 0 & 1 & 0 & -1 & 0 & 0 & 0 & 15.8716\\ 0 & 0 & 0 & 0 & 1 & 2.1121 & 2.4612 & 3.0862 & 4.0573 & 0.9212 \end{array}\right).$$

**Figure 3:**
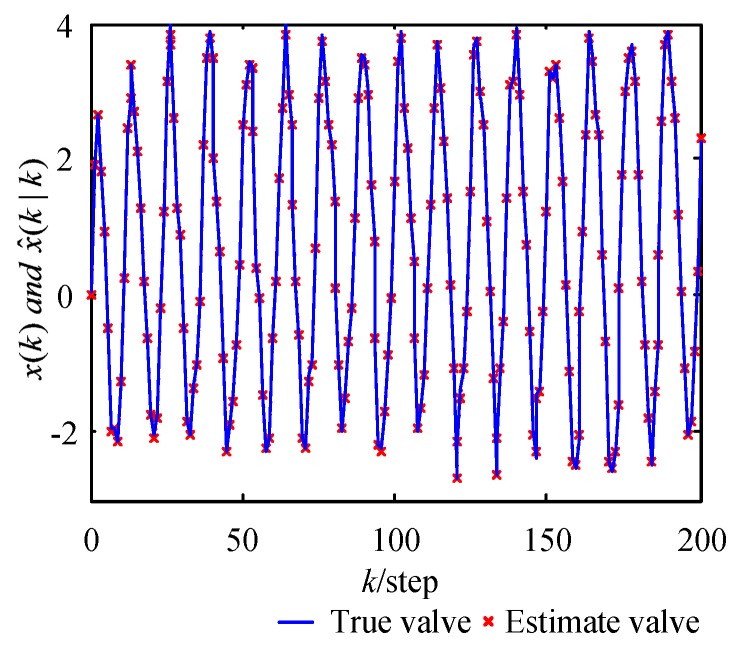
Curves of the true values and estimates using the WMF-PF algorithm based on Gauss–Hermite approximation.

Using the WMF-PF algorithm, we obtained the state estimate for the system in Equations (39) and (40). The curves of the true values and their estimates, using WMF-PF based on Gauss–Hermite approximation, are shown in Figure 3. The tracking performance is optimal.

### 6.4. Analysis

In order to compare our findings with other fusion algorithms, we introduced a kind of distributed fusion method, called a fast covariance intersection (CI) fusion algorithm [39]. Its calculation process is

(43) $${\hat{x}}_{k|k}^{\left(CI\right)}=\sum_{i=1}^{L}\omega_{i}^{}P_{CI}^{}P_{i}^{-1}{\hat{x}}_{k|k}^{\left(j\right)}$$

where ${\hat{x}}_{k|k}^{\left(CI\right)}$ is the fusion estimate by CI, ${\hat{x}}_{k|k}^{\left(j\right)},\;i=1,\cdots ,L$ are the estimates of subsystems, $P_{i}^{},\;i=1,\cdots ,L$ are the filter error variance matrices of subsystems, and

(44) $$P_{CI}^{-1}=\sum_{i=1}^{L}\omega_{i}^{}P_{i}^{-1}$$

(45) $$\omega_{j}^{}=\frac{\mathrm{trace}(P_{j}^{-1})}{\sum_{i=1}^{L}\mathrm{trace}(P_{i}^{-1})}$$

**Figure 4:**
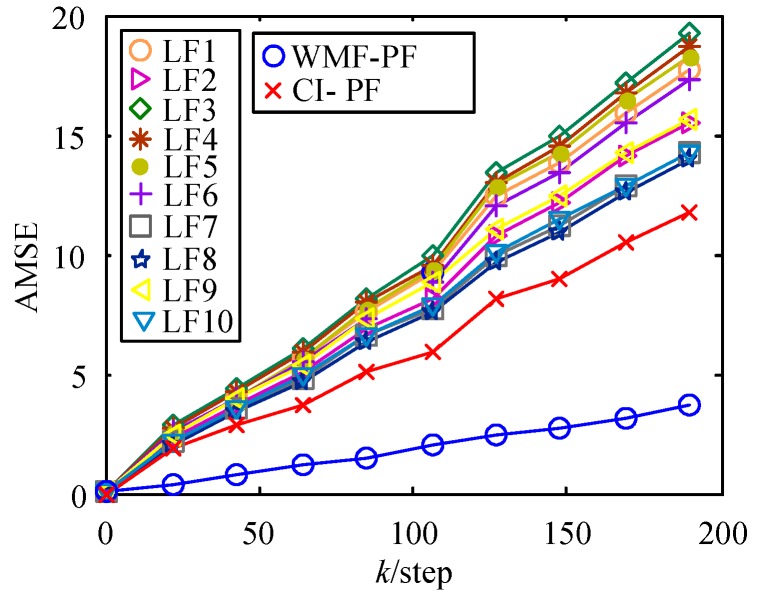
Accumulated mean square error (AMSE) curves of local particle filters (PFs), WMF-PF, and covariance intersection PF (CI-PF).

Using the CI fusion algorithm and the PF algorithm, we obtained the covariance intersection PF (CI-PF). This fusion algorithm is simple, robust, and flexible. However, its accuracy is lower than WMF. We introduced an evaluation indicator called the accumulated mean square error (AMSE) [29]:

(46) $$\mathrm{AMSE}(k)=\sum_{t=0}^{k}\frac{1}{N}\sum_{j=1}^{N}{\left[x(t)-{\hat{x}}_{j}^{}(t|t)\right]}_{}^{2}$$

where ${\hat{x}}_{j}^{}(t|t)$ is the *j*th-time Monte Carlo experiment at time *t*. The AMSE curves of local PFs (LF1–LF7), the WMF-PF, and the CI-PF are shown in Figure 4 with 20 Monte Carlo experiments. From Figure 4, the WMF-PF based on Gauss–Hermite approximation is shown to have better accuracy than local PF and CI-PF. 

**Figure 5:**
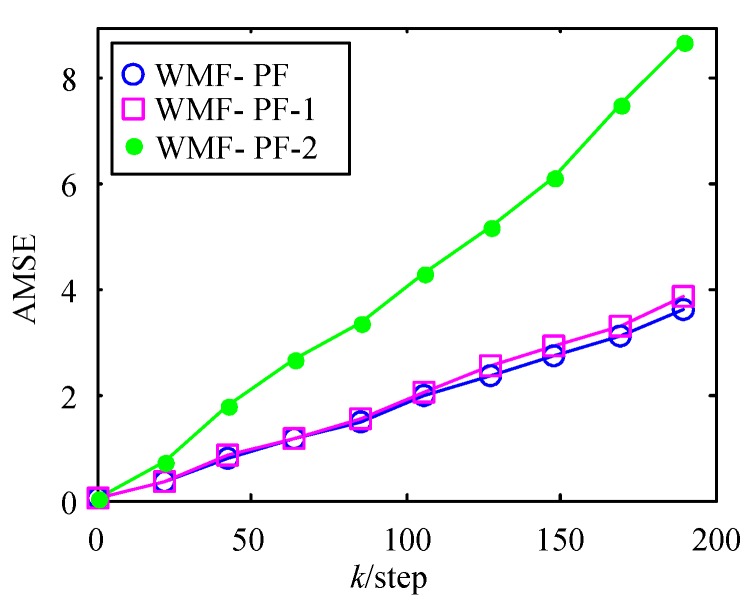
AMSE curves of WMF-PF, WMF-PF-1, and WMF-PF-2.

**Figure 6:**
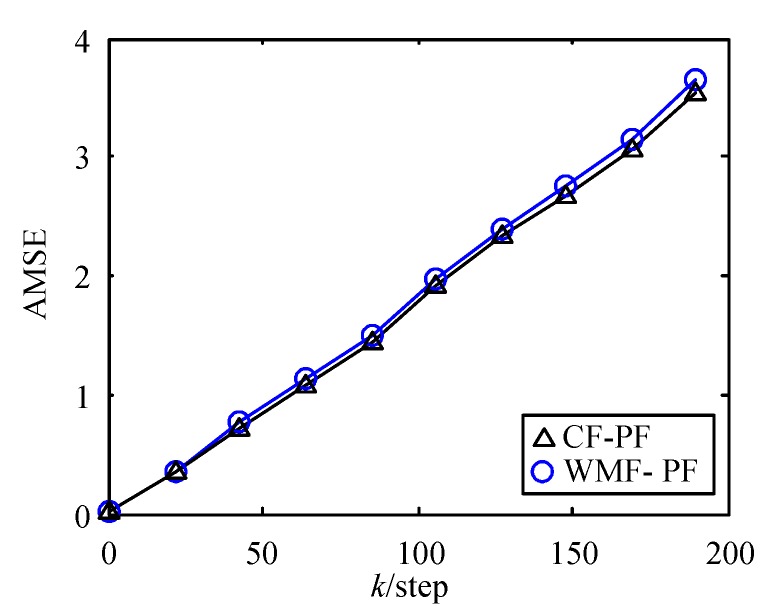
AMSE curves of WMF-PF and CF-PF.

In addition, we simulated the AMSE curves of WMF-PF under S=8,$x_{i}^{}=-3.6,\;-2.4,\;\cdots ,\;4.8$, $\Delta x_{i}^{}=1.2$, n=2, and γ=1 and WMF-PF under S=10, $\Delta x_{i}^{}=1$, n=2, and γ=0.9. To distinguish them with the above WMF-PF, we named them WMF-PF-1 and WMF-PF-2. The AMSE curves of WMF-PF, WMF-PF-1, and WMF-PF-2 are shown in Figure 5. We can see that, as the interval $\Delta x_{i}^{}$ becomes larger, the computational cost is reduced, but the estimation accuracy declines, and a reasonable parameter γ can improve the estimation accuracy. The AMSE curves of WMF-PF and CF-PF are shown in Figure 6. We can see that the accuracy of WMF-PF approximates that of CF-PF. 

Next, we compare the computational cost of WMP-PF and CF-PF. Because $H_{}^{\left(Ι\right)}$ is a 5 × 10 matrix, the time complexity of WMP-PF is $O(5_{}^{2})$. However, $H_{}^{\left(0\right)}$ is a 10×10 matrix and its time complexity is $O(10_{}^{2})$. Therefore, the computational cost of WMF-PF is obviously lower than that of CF-PF. 

In summary, the proposed WMP-PF, based on Gauss–Hermite approximation, is more accurate compared with local PFs, and has a lower computation cost when compared with CF-PF.

## 7. Conclusions

A general WMF algorithm is presented here for nonlinear multisensory systems based on the Gauss–Hermite approximation of nonlinear functions and the weighted least square method. An augmented high-dimension measurement from all sensors is compressed to a low-dimension one. Combined with the particle filter, the nonlinear weighted measurement fusion PF (WMF-PF) is presented. It can handle the nonlinear fusion estimation problem, and has reasonable accuracy and a reduced computational cost compared to centralized fusion PF. The proposed WMF algorithm can be used in systems of large-scale sensors, which can significantly reduce the computational cost and improve the real-time feature.

## Figures and Tables

**Table 1:** Mean square errors.

**Sensor Functions**	$h_{}^{\left(1\right)}\left(\cdot \right)$	$h_{}^{\left(2\right)}\left(\cdot \right)$	$h_{}^{\left(3\right)}\left(\cdot \right)$	$h_{}^{\left(4\right)}\left(\cdot \right)$	$h_{}^{\left(5\right)}\left(\cdot \right)$	$h_{}^{\left(6\right)}\left(\cdot \right)$	$h_{}^{\left(7\right)}\left(\cdot \right)$	$h_{}^{\left(8\right)}\left(\cdot \right)$	$h_{}^{\left(9\right)}\left(\cdot \right)$	$h_{}^{\left(10\right)}\left(\cdot \right)$
MSEs using Gauss–Hermite	0.0032	0.0017	0.0010	0.0014	0.0029	0.0042	0.0009	0.0013	0.0010	0.0015
MSEs using McLaughlin series	0	0	0.0258	0.0371	0.9621	1.3854	0.2286	0.3292	0.3963	0.5707
